# Arrangements of Pseudocircles: Triangles and Drawings

**DOI:** 10.1007/s00454-020-00173-4

**Published:** 2020-01-27

**Authors:** Stefan Felsner, Manfred Scheucher

**Affiliations:** Institut für Mathematik, Technische Universität, 10623 Berlin, Germany

**Keywords:** Arrangement, Pseudocircle, Triangle, Grünbaum’s conjecture, Circularizability, Tutte drawing, 52Cxx

## Abstract

A pseudocircle is a simple closed curve on the sphere or in the plane. The study of arrangements of pseudocircles was initiated by Grünbaum, who defined them as collections of simple closed curves that pairwise intersect in exactly two crossings. Grünbaum conjectured that the number of triangular cells $p_3$ in digon-free arrangements of *n* pairwise intersecting pseudocircles is at least $2n-4$. We present examples to disprove this conjecture. With a recursive construction based on an example with 12 pseudocircles and 16 triangles we obtain a family of intersecting digon-free arrangements with $p_3({\mathscr {A}})/n \rightarrow 16/11 = 1.\overline{45}$. We expect that the lower bound $p_3({\mathscr {A}}) \ge 4n/3$ is tight for infinitely many simple arrangements. It may however be true that all digon-free arrangements of *n* pairwise intersecting circles have at least $2n-4$ triangles. For pairwise intersecting arrangements with digons we have a lower bound of $p_3 \ge 2n/3$, and conjecture that $p_3 \ge n-1$. Concerning the maximum number of triangles in pairwise intersecting arrangements of pseudocircles, we show that $$p_3 \le \frac{4}{3}\left( {\begin{array}{c}n\\ 2\end{array}}\right) +O(n)$$. This is essentially best possible because there are families of pairwise intersecting arrangements of *n* pseudocircles with $$p_3 = \frac{4}{3}\left( {\begin{array}{c}n\\ 2\end{array}}\right) $$. The paper contains many drawings of arrangements of pseudocircles and a good fraction of these drawings was produced automatically from the combinatorial data produced by our generation algorithm. In the final section we describe some aspects of the drawing algorithm.

## Introduction

Arrangements of pseudocircles generalize arrangements of circles in the same vein as arrangements of pseudolines generalize arrangements of lines. The study of arrangements of pseudolines was initiated 1926 with an article of Levi [11] where he proved the ‘Extension Lemma’ and studied triangles in arrangements. Since then arrangements of pseudolines were intensively studied and the handbook article on the topic [5] lists more than 100 references.

Grünbaum [10] initiated the study of arrangements of pseudocircles. By stating a large number of conjectures he was hoping to attract the attention of researchers for the topic. The success of this program was limited and several of Grünbaum’s 45 year old conjectures remain unsettled. In this paper we report on some progress regarding conjectures involving numbers of triangles and digons in arrangements of pseudocircles.

Some of our results and new conjectures are based on a program written by the second author that enumerates all arrangements of up to seven pairwise intersecting pseudocircles. Before formally stating our main results we introduce some terminology:

An *arrangement of pseudocircles* is a collection of closed curves in the plane or on the sphere, called *pseudocircles*, with the property that the intersection of any two of the pseudocircles is either empty or consists of two points where the curves cross. An arrangement ${\mathscr {A}}$ of pseudocircles is

- *simple*, if no three pseudocircles of ${\mathscr {A}}$ intersect in a common point.
- *pairwise intersecting*, if any two pseudocircles of ${\mathscr {A}}$ have non-empty intersection. We will frequently abbreviate and just write “*intersecting*” instead of “pairwise intersecting”.
- *cylindrical*, if there are two cells of the arrangement which are separated by each of the pseudocircles.
- *digon-free*, if there is no cell of the arrangement which is incident to only two pseudocircles.

We consider the sphere to be the most natural ambient space for arrangements of pseudocircles. Consequently, we call two arrangements isomorphic if they induce homeomorphic cell decompositions of the sphere. In many cases, in particular in all our figures, arrangements of pseudocircles are embedded in the Euclidean plane, i.e., there is a distinguished outer/unbounded cell. An advantage of such a representation is that we can refer to the inner and outer side of a pseudocircle. Note that for every cylindrical arrangement of pseudocircles it is possible to choose the unbounded cell such that there is a point in the intersection of the inner discs of all pseudocircles.

In an arrangement ${\mathscr {A}}$ of pseudocircles, we denote a cell with *k* crossings on its boundary as a *k*-*cell* and let $p_k({\mathscr {A}})$ be the number of *k*-cells of ${\mathscr {A}}$. Following Grünbaum we call 2-cells *digons* and remark that some other authors call them *lenses*. 3-cells are *triangles*, 4-cells are *quadrangles*, and 5-cells are *pentagons*.

In this paper we assume that arrangements of pseudocircles are simple unless explicitly stated otherwise.

Conjecture 3.7 from Grünbaum’s monograph [10] is: *Every *(*not necessarily simple*)* digon-free arrangement of* *n* *pairwise intersecting pseudocircles has at least* $2n-4$ *triangles*. Grünbaum also provides examples of arrangements of $n\ge 6$ pseudocircles with $2n-4$ triangles.

Snoeyink and Hershberger [12] showed that the sweeping technique, which serves as an important tool for the study of arrangements of lines and pseudolines, can be adapted to work also in the case of arrangements of pseudocircles. They used sweeps to show that, in an intersecting arrangement of pseudocircles, every pseudocircle is incident to two cells which are digons or triangles on either side. Therefore, $2p_2 + 3p_3 \ge 4n$ which implies that every intersecting digon-free arrangement of *n* pseudocircles has at least 4*n*/3 triangles.

Felsner and Kriegel [6] observed that the bound from [12] also applies to non-simple intersecting digon-free arrangements and gave examples of arrangements showing that the bound is tight on this class for infinitely many values of *n*. These examples disprove the conjecture in the non-simple case.

In Sect. 2, we give counterexamples to Grünbaum’s conjecture [10, Conjecture 3.7] which are simple. With a recursive construction based on an example with 12 pseudocircles and 16 triangles we obtain a family of digon-free intersecting arrangements with $p_3/n \xrightarrow {n \rightarrow \infty } 16/11 = 1.\overline{45}$. We then replace Grünbaum’s conjecture by Conjecture 2.7: *The lower bound* $p_3({\mathscr {A}}) \ge 4n/3$ *is tight for infinitely many simple arrangements*.

A specific arrangement ${\mathscr {N}}_6^\Delta$ of six pseudocircles of eight triangles is interesting in this context. The arrangement ${\mathscr {N}}_6^\Delta$ has no representation with circles, two different proofs for the non-circularizablility of ${\mathscr {N}}_6^\Delta$ have been given in [8] and [9]. The arrangement ${\mathscr {N}}_6^\Delta$ appears as a subarrangement in all known simple, intersecting, digon-free arrangements with $p_3 < 2n-4$. This motivates the question, whether indeed Grünbaum’s conjecture is true when restricted to intersecting arrangements of circles, see Conjecture 2.2. In Sect. 2.1 we discuss arrangements with digons. We give an easy extension of the argument of Snoeyink and Hershberger [12] to show that these arrangements contain at least 2*n*/3 triangles. All intersecting arrangements known to us have at least $n-1$ triangles and therefore our Conjecture 2.10 is that $n-1$ is a tight lower bound for intersecting arrangements with digons.

In Sect. 3 we study the maximum number of triangles in arrangements of *n* pseudocircles. We show an upper bound of order $2n^2/3+O(n)$. For the lower bound construction we glue two arrangements of *n* pseudolines into an arrangement of *n* pseudocircles. Since respective arrangements of pseudolines are known, we obtain arrangements of pseudocircles with $2n(n-1)/3$ triangles for $n \equiv 0,4 \pmod 6$.

The paper contains many drawings of arrangements of pseudocircles and a good fraction of these drawings was produced automatically from the combinatorial data produced by the generation algorithm. In Sect. 4 we describe some aspects of the drawing algorithm which is based on iterative calls to a Tutte embedding a.k.a. spring embedding with adapting weights on the edges.

## Intersecting Arrangements with Few Triangles

The main result of this section is the following theorem, which disproves Grünbaum’s conjecture.

### Theorem 2.1

The minimum number of triangles in digon-free intersecting arrangements of *n* pseudocircles is

(i) 8 for $$3\le n \le 6$$.
(ii) $$\lceil {4}n/{3}\rceil $$ for $$6 \le n \le 14$$.
(iii) $<{16}n/{11}$ for all $n =11k+1$ with $k\in \mathbb {N}$.

**Fig. 1:**
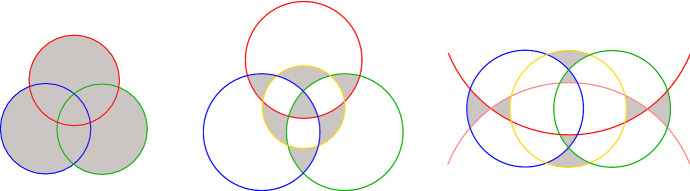
Digon-free intersecting arrangements of $n=3,4,5$ circles with $p_3=8$ triangles. Triangles (except the outer face) are colored gray

**Fig. 2:**
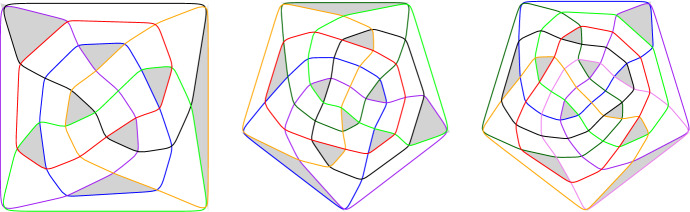
Digon-free intersecting arrangements of $n=6,7,8$ pseudocircles with 8, 10, 11 triangles, respectively.The arrangement of $n=6$ pseudocircles on the left-hand side is named ${\mathscr {N}}_6^\Delta$

Figures 1 and 2 show intersecting arrangements with the minimum number of triangles for up to eight pseudocircles. We remark that, in total, there are three non-isomorphic intersecting arrangements of $n=8$ pseudocircles with $p_3 = 11$ triangles, these are the smallest counterexamples to Grünbaum’s conjecture (cf. Lemma 2.3). We refer to our website [7] for further examples.

The basis for Theorem 2.1 was laid by exhaustive computations, which generated all intersecting arrangements of up to $n=7$ pseudocircles. Starting with the unique intersecting arrangement of two pseudocircles, our program recursively inserted pseudocircles in all possible ways. From the complete enumeration, we know the minimum number of triangles for $n\le 7$. In the range from 8 to 14, we had to iteratively use arrangements of *n* pseudocircles with a small number of triangles and digons to generate arrangements of $n+1$ pseudocircles with the same property. Using this strategy, we found intersecting arrangements with $\lceil 4n/3\rceil$ triangles for all *n* in this range. The corresponding lower bound $p_3({\mathscr {A}}) \ge 4n/3$ is known from [12].

The approach, which we had used to tackle arrangements of up to $n=14$ pseudocircles, made the complete enumerating of all arrangements obsolete. However, since enumeration and counting is also much of interest in the context of arrangements we decided to move the corresponding results to [9], where we investigate (not necessarily intersecting) arrangements and focus on circularizability. The arrangements and more information can be also be found on our companion website [7].

Another result which we obtained from our computer search is the following: the triangle-minimizing example for $n=6$ is unique, i.e., there is a unique intersecting arrangement ${\mathscr {N}}_6^\Delta$ of six pseudocircles with eight triangles. In [8] and [9] we gave different proofs for the non-circularizability of ${\mathscr {N}}_6^\Delta$. Since the arrangement ${\mathscr {N}}_6^\Delta$ appears as a subarrangement of all arrangements with less than $2n-4$ triangles known to us, the following weakening of Grünbaum’s conjecture might be true.

### Conjecture 2.2

(Weak Grünbaum Conjecture) Every digon-free intersecting arrangement of *n* circles has at least $2n-4$ triangles.

If this conjecture were true, it would imply a simple non-circularizability criterion for intersecting arrangements: Any arrangement with $p_3 < 2n-4$ could directly be classified as non-circularizable.

So far we know that this conjecture is true for all $n\le 9$. The claim, that we have checked all intersecting arrangements with $p_3({\mathscr {A}}) < 2n-4$ in this range, is justified by the following lemma, which restricts the pairs $(p_2,p_3)$ for which there can exist arrangements of *n* pseudocircles whose extensions have $p_3({\mathscr {A}}) < 2n-4$. For example, to get all digon-free intersecting arrangements of $n=9$ pseudocircles with $p_3 \le 13$ triangles, we only had to extend intersecting arrangements of $n=7$ and $n=8$ pseudocircles with $p_3 + 2p_2 \le 13$ triangles.

### Lemma 2.3

Let ${\mathscr {A}}$ be an intersecting arrangement of pseudocircles. Then for every subarrangement ${\mathscr {A}}'$ of ${\mathscr {A}}$ we have

$$\begin{aligned} p_3({\mathscr {A}}')+ 2p_2({\mathscr {A}}') \le p_3({\mathscr {A}}) + 2p_2({\mathscr {A}}). \end{aligned}$$

### Proof

We show the statement for a subarrangement ${\mathscr {A}}'$ in which one pseudocircle *C* is removed from ${\mathscr {A}}$. The inequality then follows by iterating the argument. The arrangement ${\mathscr {A}}'$ partitions the pseudocircle *C* into arcs. Reinsert these arcs one by one.

Consider a triangle of ${\mathscr {A}}'$. After adding an arc, one of the following cases occurs: (1) the triangle remains untouched, or (2) the triangle is split into a triangle and a quadrangle, or (3) a digon is created in the region of the triangle.

Now consider a digon of ${\mathscr {A}}'$. After adding an arc, one of the following cases occurs: (1) the digon remains untouched, or (2) there is a new digon inside this digon, or (3) the digon has been split into two triangles.$\square$

Levi [11] has shown that every arrangement of pseudolines in the real projective plane has at least *n* triangles. Since arrangements of great-(pseudo)circles are in bijection to arrangements of (pseudo)lines (the bijection is explained in Sect. 3.1), it directly follows that every arrangement of great-pseudocircles has at least 2*n* triangles. The next theorem applies the same idea to a superclass of great-pseudocircle arrangements. We think of the theorem as support of the Weak Grünbaum Conjecture (Conjecture 2.2).

### Theorem 2.4

Let ${\mathscr {A}}$ be an intersecting arrangement of *n* pseudocircles such that there is a pseudocircle *C* in ${\mathscr {A}}$ that separates the two intersection points $C' \cap C''$ of any other two pseudocircles $C'$ and $C''$ in ${\mathscr {A}}$. Then the number of triangles in ${\mathscr {A}}$ is at least 2*n*.

### Proof

Since, for every two pseudocircles $C'$ and $C''$ distinct from *C*, the two intersection points of $C' \cap C''$ are separated by the pseudocircle *C*, the pseudocircle *C* “partitions” the arrangement ${\mathscr {A}}$ into two projective arrangements of *n* pseudolines which lie in the two respective hemispheres. According to Levi [11], there are at least *n* triangles in each of the two arrangements, thus the original arrangement ${\mathscr {A}}$ contains at least 2*n* triangles. $\square$

Felsner and Kriegel [6] have shown that every arrangement of *n* pseudolines in the Euclidean plane has at least $n-2$ triangles. This can again be turned into a result about triangles in arrangements of pseudocircles.

### Theorem 2.5

Let ${\mathscr {A}}$ be an intersecting arrangement of *n* pseudocircles. If ${\mathscr {A}}$ can be extended by another pseudocircle *C* so that the pseudocircle *C* separates the two intersection points $C' \cap C''$ of any other two pseudocircles $C'$ and $C''$, then the number of triangles in the original arrangement ${\mathscr {A}}$ is at least $2n-4$.

### Proof

Since, for every two pseudocircles $C'$ and $C''$ distinct from *C*, the two intersection points of $C' \cap C''$ are separated by *C*, the pseudocircle *C* splits the arrangement ${\mathscr {A}}$ into two Euclidean arrangements of *n* pseudolines which lie in the two respective hemispheres. According to Felsner and Kriegel [6], there are at least $n-2$ triangles in each of the two arrangements. Since the extending pseudocircle *C* (which can be considered as the line at infinity in the respective Euclidean pseudoline arrangements) is not incident to any of these triangles, the arrangement ${\mathscr {A}}$ contains at least $2n-4$ triangles. $\square$

**Fig. 3:**
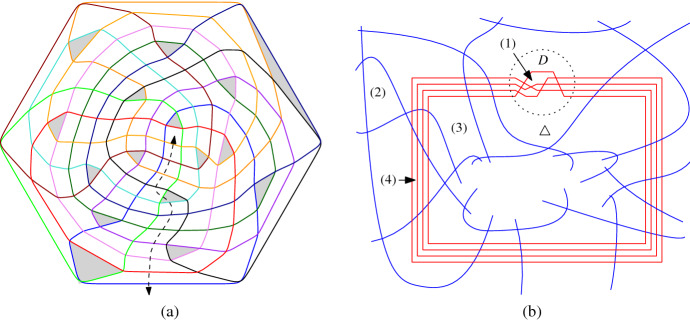
(**a**) The digon-free intersecting arrangement ${\mathscr {A}}_{12}$ of 12 pseudocircles with exactly 16 triangles. The dashed curve intersects every pseudocircle exactly once. (**b**) An illustration of the construction in Lemma 2.6. Pseudocircles of ${\mathscr {A}}$ and ${\mathscr {B}}$ are drawn red and blue, respectively.

We now prepare for the proof of Theorem 2.1 (iii), for which we construct a family of (non-circularizable) intersecting arrangements of *n* pseudocircles with less than 16*n*/11 triangles. The basis of the construction is the arrangement ${\mathscr {A}}_{12}$ of 12 pseudocircles with 16 triangles shown in Fig. 3 (a). This arrangement will be used iteratively for a ‘merge’ as described by the following lemma.

### Lemma 2.6

Let ${\mathscr {A}}$ and ${\mathscr {B}}$ be digon-free intersecting arrangements of $n_{\mathscr {A}}\ge 3$ and $n_{\mathscr {B}}\ge 3$ pseudocircles, respectively. If there is a simple curve $P_{\mathscr {A}}$ that

1. intersects every pseudocircle of ${\mathscr {A}}$ exactly once,
2. contains no vertex of ${\mathscr {A}}$,
3. traverses $\tau \ge 1$ triangles of ${\mathscr {A}}$, and
4. forms $\delta$ triangles with pairs of pseudocircles from ${\mathscr {A}}$,

then there is a digon-free intersecting arrangement ${\mathscr {C}}$ of $n_{\mathscr {A}}+n_{\mathscr {B}}-1$ pseudocircles with $p_3({\mathscr {C}}) = p_3({\mathscr {A}}) + p_3({\mathscr {B}}) + \delta - \tau -1$ triangles.

We remark that condition (1) from the statement of Lemma 2.6 asserts that ${\mathscr {A}}$ is cylindrical. Moreover, if ${\mathscr {B}}$ is cylindrical, then also ${\mathscr {C}}$ is cylindrical.

### Proof

Take a drawing of ${\mathscr {A}}$ and make a hole in the two cells which contain the ends of $P_{\mathscr {A}}$. This yields a drawing of ${\mathscr {A}}$ on a cylinder such that none of the pseudocircles is contractible. The path $P_{\mathscr {A}}$ connects the two boundaries of the cylinder. In fact, the existence of a path with the properties of $P_{\mathscr {A}}$ characterizes cylindrical arrangements.

Stretch the cylindrical drawing so that it becomes a narrow belt, where all intersections of pseudocircles take place in a small disk, which we call *belt-buckle*. This drawing of ${\mathscr {A}}$ is called a *belt drawing*. The drawing of the red subarrangement in Fig. 3 (b) shows a belt drawing.

Choose a triangle $\triangle$ in ${\mathscr {B}}$ and a pseudocircle *B* which is incident to $\triangle$. Let *b* be the *edge* of *B* on the boundary of $\triangle$. Specify a disk *D*, which is traversed by *b* and disjoint from all other edges of ${\mathscr {B}}$. Now replace *B* by a belt drawing of ${\mathscr {A}}$ in a small neighborhood of *B* such that the belt-buckle is drawn within *D*; see Fig. 3 (b).

The arrangement ${\mathscr {C}}$ obtained from *merging* ${\mathscr {A}}$ and ${\mathscr {B}}$, as we just described, has $n_{\mathscr {A}}+ n_{\mathscr {B}}- 1$ pseudocircles. Moreover if ${\mathscr {A}}$ and ${\mathscr {B}}$ are digon-free/intersecting, then ${\mathscr {C}}$ has the same property. Most of the cells *c* of ${\mathscr {C}}$ are of one of the following four types:

1. All boundary edges of *c* belong to pseudocircles of ${\mathscr {A}}$.
2. All boundary edges of *c* belong to pseudocircles of ${\mathscr {B}}$.
3. All but one of the boundary edges of *c* belong to pseudocircles of ${\mathscr {B}}$ and the remaining edge belongs to ${\mathscr {A}}$. (These cells correspond to cells of ${\mathscr {B}}$ with a boundary edge on *B*.)
4. Quadrangular cells, whose boundary edges alternatingly belong to ${\mathscr {A}}$ and ${\mathscr {B}}$.

From the cells of ${\mathscr {B}}$, only $\triangle$ and the other cell containing *b* (which is not a digon since ${\mathscr {B}}$ is digon-free) have not been taken into account. In ${\mathscr {C}}$, the corresponding two cells have at least two boundary edges from ${\mathscr {B}}$ and at least two from ${\mathscr {A}}$. Consequently, neither of the two cells are triangles. The remaining cells of ${\mathscr {C}}$ are bounded by pseudocircles from ${\mathscr {A}}$ together with one of the two bounding pseudocircles of $\triangle$ other than *B*. These two pseudocircles cross through ${\mathscr {A}}$ following the path prescribed by $P_{\mathscr {A}}$. There are $\delta$ triangles among these cells, but $\tau$ of these are obtained because $P_{\mathscr {A}}$ traverses a triangle of ${\mathscr {A}}$. Among cells of ${\mathscr {C}}$ of types (1) to (4) all the triangles have a corresponding triangle in ${\mathscr {A}}$ or ${\mathscr {B}}$. But $\triangle$ is a triangle of ${\mathscr {B}}$ which does not occur in this correspondence. Hence, there are $p_3({\mathscr {A}}) + p_3({\mathscr {B}}) +\delta -\tau -1$ triangles in ${\mathscr {C}}$. $\square$

### Proof of Theorem 2.1(iii)

We use ${\mathscr {A}}_{12}$, the arrangement shown in Fig. 3 (a), in the role of ${\mathscr {A}}$ for our recursive construction. The dashed path in the figure is used as $P_{\mathscr {A}}$ with $\delta =2$ and $\tau =1$. Starting with ${\mathscr {C}}_1={\mathscr {A}}_{12}$ and defining ${\mathscr {C}}_{k+1}$ as the merge of ${\mathscr {C}}_k$ and ${\mathscr {A}}_{12}$, we construct a sequence $\{{\mathscr {C}}_{k}\}_{k \in \mathbb {N}}$ of digon-free intersecting arrangements of $n({\mathscr {C}}_{k}) = 11k+1$ pseudocircles with $p_3({\mathscr {C}}_{k}) = 16k$ triangles. The fraction $16k/(11k+1)$ is increasing with *k* and converges to $16/11 = 1.\overline{45}$ as *n* goes to $\infty$. $\square$

We remark that using other arrangements from Theorem 2.1 (ii) (which also admit a path with $\delta =2$ and $\tau =1$) in the recursion, we obtain intersecting arrangements with $p_3 = \lceil {16}n/{11} \rceil$ triangles for all $n \ge 6$.

Since the lower bound $\lceil {4}n/{3}\rceil$ is tight for $6 \le n \le 14$, we believe that the following is true:

### Conjecture 2.7

There are digon-free intersecting arrangements ${\mathscr {A}}$ of *n* pseudocircles with $p_3({\mathscr {A}}) = \lceil 4n/3 \rceil$ for infinitely many values of *n*.

### Intersecting Arrangements with Digons

**Fig. 4:**
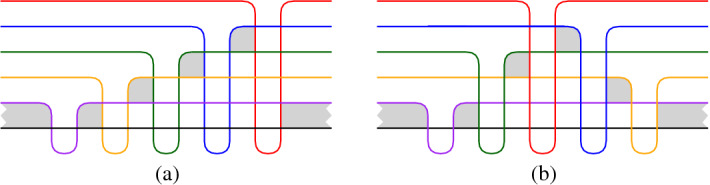
Intersecting arrangements of *n* pseudocircles with *n* digons and $n-1$ triangles

We know intersecting arrangements of $n \ge 3$ pseudocircles with digons and only $n-1$ triangles. The examples depicted in Fig. 4 are part of an infinite family of such arrangements. As illustrated, the intersection order with the black circle determines the arrangement. In fact, it is easy to see that $2^{n-3}$ different arrangements are possible: Starting with the black, the purple, and the yellow pseudocircles (which give a unique arrangement), each further pseudocircle has its finger placed either immediately to the left or immediately to the right of the previous finger. Figures 4 (a) and 4 (b) illustrate the finger-insertion-sequence “right–right–right–...” and “left–right–left–...”, respectively.

Using ideas based on sweeps (cf. [12]), we can show that every pseudocircle is incident to at least two triangles. This implies the following theorem:

#### Theorem 2.8

Every intersecting arrangement of $n \ge 3$ pseudocircles has at least 2*n*/3 triangles.

The proof of the theorem is based on the following lemma:

#### Lemma 2.9

Let *C* be a pseudocircle in an intersecting arrangement of $n \ge 3$ pseudocircles. Then all digons incident to *C* lie on the same side of *C*.

#### Proof

Consider a pseudocircle $C'$ that forms a digon $D'$ with *C* that lies, say, “inside” *C*. If $C''$ also forms a digon $D''$ with *C*, then $C''$ has to cross $C'$ in the exterior of *C*. Hence $D''$ also has to lie “inside” *C*. Consequently, all digons incident to *C* lie on the same side of *C*. $\square$

#### Proof of Theorem 2.8

Let ${\mathscr {A}}$ be an intersecting arrangement and consider a drawing of ${\mathscr {A}}$ in the plane. Snoeyink and Hershberger [12] have shown that starting with any circle *C* from ${\mathscr {A}}$ the outside of *C* can be swept with a closed curve $\gamma$ until all of the arrangement is inside of $\gamma$. During the sweep $\gamma$ intersects every pseudocircle from ${\mathscr {A}}$ at most twice. The sweep uses two types^1^ of moves to make progress:

1. *take a crossing*, in [12] this is called ‘pass a triangle’;
2. *leave a pseudocircle*, this is possible when $\gamma$ and some pseudocircle form a digon which is on the outside of $\gamma$, in [12] this is called ‘pass a hump’.

**Fig. 5:**
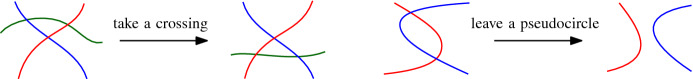
An illustration of the two types of moves which are possible in the proof of Theorem 2.8. The blue curve is $\gamma$. The interior of $\gamma$ is left of the shown part of the curve

Figure 5 gives an illustration of the two possible types of moves.

Let *C* be a pseudocircle of ${\mathscr {A}}$. By the previous lemma, all digons incident to *C* lie on the same side of *C*. Redraw ${\mathscr {A}}$ so that all digons incident to *C* are inside *C*. The first move of a sweep starting at *C* has to take a crossing, and hence, there is a triangle $\triangle$ incident to *C*. Redraw ${\mathscr {A}}$ such that $\triangle$ becomes the unbounded face. Again consider a sweep starting at *C*. The first move of this sweep reveals a triangle $\triangle '$ incident to *C*. Since $\triangle$ is not a bounded triangle of the new drawing we have $\triangle \ne \triangle '$, and hence, *C* is incident to at least two triangles. The proof is completed by double counting the number of incidences of triangles and pseudocircles. $\square$

Since for $3 \le n \le 7$ every intersecting arrangement has at least $n-1$ triangles, we believe that the following is true:

#### Conjecture 2.10

Every intersecting arrangement of $n \ge 3$ pseudocircles has at least $n-1$ triangles.

**Fig. 6:**
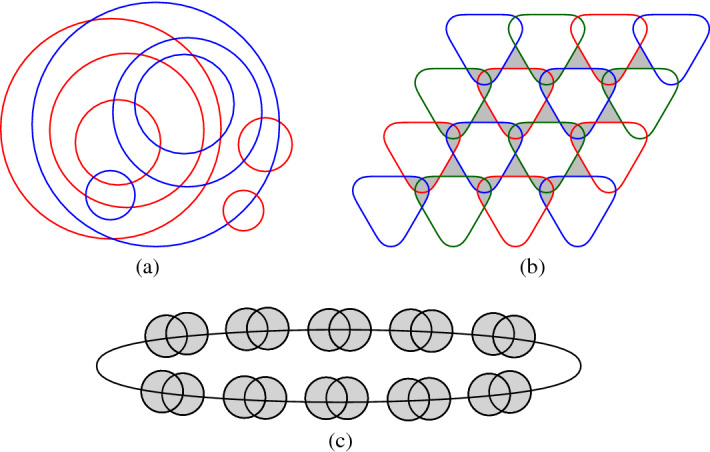
Non-intersecting arrangements (**a**) with no triangles, (**b**) with a triangle-cell-ratio of $5/6+O(1/\sqrt{n})$, and (**c**) with only two non-triangular cells, i.e., with a triangle-cell-ratio of $1+O(1/n)$

If the arrangement is not required to be intersecting, then the proof of Lemma 2.9 fails. Indeed, if the intersection graph of the arrangement is bipartite, then all faces are of even degree, in particular, there are no triangles; see Fig. 6 (a).

## Maximum Number of Triangles

**Fig. 7:**
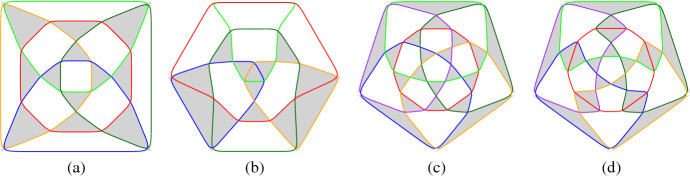
(**a**) and (**b**) show arrangements of $n=5$ pseudocircles. The first one is digon-free and has 12 triangles and the second one has 13 triangles and one digon. (**c**) and (**d**) show arrangements of $n=6$ with 20 triangles. The arrangement in (c) is the skeleton of the icosidodecahedron

**Fig. 8:**
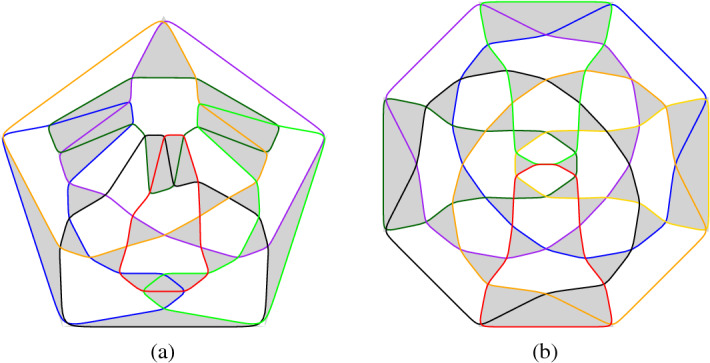
(**a**) An arrangement of $n=7$ pseudocircles with 29 triangles. (**b**) An arrangement of $n=8$ pseudocircles with 38 triangles

Regarding the maximum number of triangles the complete enumeration^2^ provides precise data for $n\le 8$. Moreover, we used heuristics to generate examples with many triangles for larger *n*. Table 1 summarizes our results and Figs. 7 and 8 show intersecting arrangements of $n=5,6,7,8$ pseudocircles with the maximal number of triangles; further arrangements are available on our website [7].

**Table 1:** Maximum number of triangles in intersecting arrangements of *n* pseudocircles

*n*	2	3	4	5	6	7	8	9	10
Simple	0	8	8	13	20	29	38	$\ge 48$	$\ge 60$
Digon-free	–	8	8	12	20	29	38	$\ge 48$	$\ge 60$
$$\bigl \lfloor \tfrac{4}{3}\left( {\begin{array}{c}n\\ 2\end{array}}\right) \bigr \rfloor $$	1	4	8	13	20	28	37	48	60

In the next subsection we show that asymptotically the contribution of edges that are incident to two triangles is neglectable. The last subsection gives a construction of intersecting arrangements which show that $$\bigl \lfloor \tfrac{4}{3}\left( {\begin{array}{c}n\\ 2\end{array}}\right) \bigr \rfloor $$ is attained for infinitely many values of *n*.

Recall that we only study simple intersecting arrangements. Grünbaum [10] also looked at non-simple arrangements. His Figs. 3.30, 3.31, and 3.32 show drawings of simplicial arrangements that have $n=7$ with $p_3= 32$, $n=8$ with $p_3= 50$, and $n=9$ with $p_3= 62$, respectively. Hence, non-simple arrangements can have more triangles.

### Theorem 3.1

Every intersecting arrangement ${\mathscr {A}}$ of pseudocircles fulfills $$p_3({\mathscr {A}}) \le \frac{4}{3}\left( {\begin{array}{c}n\\ 2\end{array}}\right) +O(n)$$.

### Proof

Let ${\mathscr {A}}$ be an intersecting arrangement of $n\ge 4$ pseudocircles. We view ${\mathscr {A}}$ as a 4-regular plane graph, i.e., the set *X* of crossings is the vertex set and edges are the segments which connect consecutive crossings on a pseudocircle.

### Claim 3.2

No crossing is incident to four triangular cells.

**Fig. 9:**
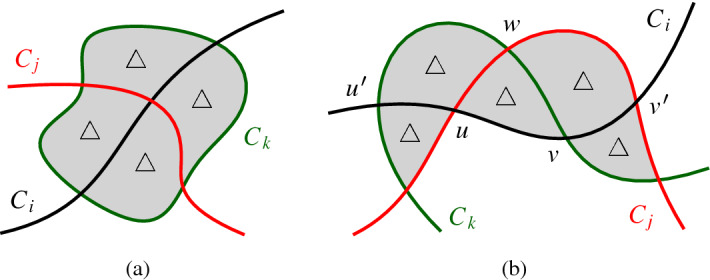
Illustrations of the proof of Claim 3.2 and Claim 3.3

Assume that a crossing *u* of $C_i$ and $C_j$ is incident to four triangular cells. Then there is a pseudocircle $C_k$ which bounds those four triangles, see Fig. 9 (a). Now $C_k$ only intersects $C_i$ and $C_j$. This, however, is impossible because $n \ge 4$ and ${\mathscr {A}}$ is intersecting.   $\square$

Let $X' \subseteq X$ be the set of crossings of ${\mathscr {A}}$ that are incident to three triangular cells. Our aim is to show that $|X'|$ is small, in fact $|X'| \in O(n)$. When this is shown we can bound the number of triangles in ${\mathscr {A}}$ as follows: Under the assumption that $|X'| \in O(n)$, the number of triangles incident to a crossing in $X'$ clearly is in *O*(*n*). Now let $Y = X\setminus X'$. Each of the remaining triangles is incident to three elements of *Y* and each crossing of *Y* is incident to at most two triangles. Hence, there are at most $2|Y|/3 + O(n)$ triangles. Since $|Y| \le |X| = n(n-1)$ we obtain the bound claimed in the statement of the theorem.

To show that $|X'|$ is small we need some preparation.

### Claim 3.3

Two adjacent crossings *u*, *v* in $X'$ share two triangles.

Since *u* and *v* are both incident to three triangles, there is at least one triangle $\triangle$ incident to both of them. Assume for a contradiction that the other cell which is incident to the segment *uv* is not a triangle. Let $C_i,C_j,C_k$ be the three pseudocircles such that *u* is a crossing of $C_i$ and $C_j$, *v* is a crossing of $C_i$ and $C_k$, and $\triangle$ is bounded by $C_i,C_j,C_k$; see Fig. 9 (b). We denote the third vertex of $\triangle$ by *w* and note that *w* is a crossing of $C_j$ and $C_k$.

Since *u* is incident to three triangles, the segment *uw* bounds another triangle, which is again defined by $C_i,C_j,C_k$. Let $u'$ be the third vertex incident to that triangle. Similarly, the segment *vw* is incident to another triangle which is also defined by $C_i,C_j,C_k$, and has a third vertex $v'$.

Again, by the same argument, the segments $uu'$ and $vv'$, respectively, are both incident to another triangle. However, this is impossible as the two circles $C_j$ and $C_k$ intersect three times. Thus both faces incident to segment *uv* are triangles. $\square$

### Claim 3.4

Let *u*, *v*, *w* be three distinct crossings in $X'$. If *u* is adjacent to both *v* and *w*, then *v* is adjacent to *w*.

Since *u* is incident to three triangles and the segments *uv* and *uw* are both incident to two triangles, there is a triangle $\triangle$ with corners *u*, *v*, *w*. This triangle shows that *u*, *v*, and *w* are adjacent to each other. $\square$

**Fig. 10:**
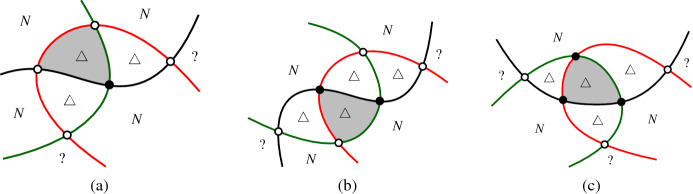
An illustration of the configurations of crossings in $X'$. In this figure $\triangle$ marks a triangle, “*N*” marks a *k*-cell with $k \ge 4$ (“neither a triangle, nor a digon”), “?” marks an arbitrary cell. Crossings with three incident triangles are shown as black vertices (these are the crossings in $X'$)

Claim 3.4 implies that each connected component of the graph induced by $X'$ is a complete graph. It is easy to see that a $K_4$ induced by $X'$ is impossible, and therefore, all components induced by $X'$ are either singletons, edges, or triangles. Figure 10 shows the local structure of the arrangement around components of these three types.

To show that $|X'|$ is small, we are going to trade crossings of $X'$ with digons and then refer to a result of Agarwal et al. [1]. They have shown that the number of digons in intersecting arrangements of pseudocircles is at most linear in *n*.

**Fig. 11:**
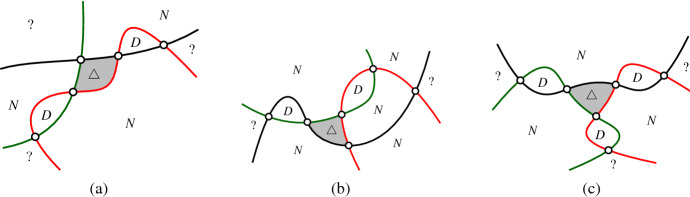
The configurations in (**a**, **b**), and (**c**) are obtained by flipping the gray triangle in the configuration from Fig. 10 (a), (b), and (c), respectively. The digons created by the flip are marked “*D*”

To convert crossings of $X'$ into digons we use *triangle flips*. Each of the configurations shown in Fig. 10 has a gray triangle. By flipping these triangles we obtain the configurations shown in Fig. 11. These so-obtained configurations have at least as many new digons as the original configurations contain crossings in $X'$. It may be that the flip creates new triangles and even new vertices which are incident to three triangles. However, the flips never remove digons.

Therefore, thanks to the result from [1] we can make no more than *O*(*n*) flips before all the crossings are incident to at most two triangles. This finishes the proof of Theorem 3.1. $\square$

In the proof of Theorem 3.1, we have used flips to trade segments incident to two triangles against digons. It can be shown that at most one component of the graph induced by $X'$ is a $K_3$. The proof of this fact is omitted here since it does not improve the bound given in the theorem. Having used a bound on the number of digons we recall that Grünbaum conjectures that $p_2 \le 2n-2$ holds for intersecting arrangements.

Since intersecting arrangements have $$2\left( {\begin{array}{c}n\\ 2\end{array}}\right) +2$$ faces, we can also rewrite the statement of Theorem 3.1: at most $\frac{2}{3}+O\bigl (\frac{1}{n}\bigr )$ of all cells of an intersecting arrangement are triangles. For $n=7$ there exist arrangements with $$29=\frac{4}{3}\left( {\begin{array}{c}7\\ 2\end{array}}\right) +1$$ triangles. It would be interesting to know what the precise maximum value of $p_3$ for *n* large.

For non-intersecting arrangements the arguments from the proof of Theorem 3.1 do not work. Figure 6 (c) shows an arrangement where all but two cells are triangles. However, if each pseudocircle is required to intersect at least three other pseudocircles, then we can proceed similar and show that the triangle-cell-ratio is at most $5/6+O(1/n)$. In fact, Fig. 6 (b) shows a construction with triangle-cell-ratio $5/6+O(1/\sqrt{n})$. $\square$

### Theorem 3.5

Let ${\mathscr {A}}$ be an arrangement of *n* pseudocircles where every pseudocircle intersects at least three other pseudocircles. Then the triangle-cell-ratio is at most $5/6+O(1/n)$.

### Proof

We proceed as in the proof of Theorem 3.1. In fact, as the “intersecting” property was only used to bound the number of digons, Claims 3.2–3.4 hold also in this less restrictive setting.

From Claims 3.2–3.4 we have learned that every vertex from $X'$ has at least two neighbors from $X \setminus X'$. The following claim will help us to show $|X'| \le |X \setminus X'|$.

### Claim 3.6

Every vertex from $X \setminus X'$ has at most two neighbors from $X'$.

Suppose for a contradiction that a vertex $v \in X \setminus X'$ has (at least) three neighbors *x*, *y*, *z* from $X'$. Since *x*, *y*, *z* each have three incident triangular faces and since $v \not \in X'$, two of the neighboring faces of *v* are triangles. In particular, those two triangular faces are not adjacent as otherwise *x*, *y*, *z* would lie in the same component of $G[X']$ and have the same neighbor *v* – which is impossible.

**Fig. 12:**
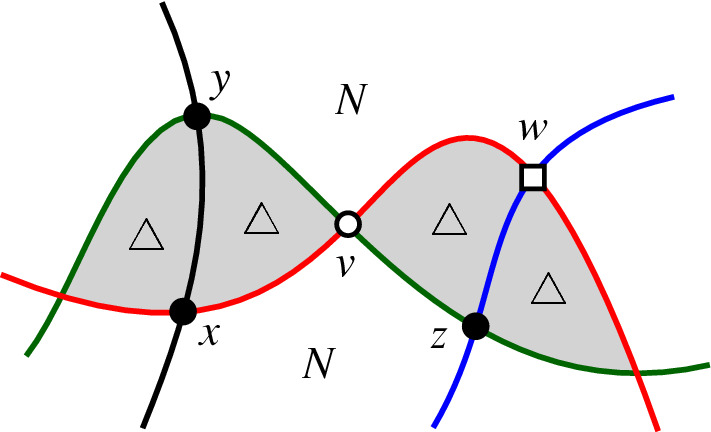
An illustration of the proof of Claim 3.6

Without loss of generality, we assume that *xy* is an edge and that *z* forms an edge with the fourth neighbor of *v*, which we denote by *w*. Since *x* is incident to a non-triangular face (which is also incident to *v*), the edge *xy* bounds another triangle. The same argument shows that *zw* bounds another triangle, and therefore, the two pseudocircles passing through *v* intersect three times—a contradiction; see Fig. 12. This finishes the proof of Claim 3.6. $\square$

We can now discharge 1/2 from every vertex of $X'$ to its neighbors from $X \setminus X'$ and, by Claim 3.6, count at most 1 at each of the vertices from $X \setminus X'$. Therefore, $|X'| \le |X \setminus X'|$ holds. By counting the face-vertex-incidences we get

$$\begin{aligned} p_3 \le \frac{3|X'|+2|X \setminus X'|}{3}\le \frac{5|X|}{6} , \end{aligned}$$

and since the number of faces equals $|X|+2$, this completes the proof of Theorem 3.5. $\square$

### Constructions Using Arrangements of Pseudolines

Great-circles on the sphere are a well-known model for projective arrangements of lines. Antipodal pairs of points on the sphere correspond to points of the projective plane. Hence, the great-circle arrangement corresponding to a projective arrangement ${\mathscr {A}}$ of lines has twice as many vertices, edges, and faces of every type as ${\mathscr {A}}$. The same idea can be applied to projective arrangements of pseudolines. If ${\mathscr {A}}$ is a projective arrangement of pseudolines, take a drawing of ${\mathscr {A}}$ in the unit disk *D* such that every line $\ell$ of ${\mathscr {A}}$ connects two antipodal points of *D*. Project *D* to the upper hemisphere of a sphere *S*, so that the boundary of *D* becomes the equator of *S*. Use a projection through the center of *S* to copy the drawing from the upper hemisphere to the lower hemisphere of *S*. By construction the two copies of each pseudoline from ${\mathscr {A}}$ join together to form a pseudocircle. The collection of these pseudocircles yields an intersecting arrangement of pseudocircles on the sphere with twice as many vertices, edges, and faces of every type as ${\mathscr {A}}$. Arrangements of pseudocircles obtained by this construction have a special property:

- If three pseudocircles *C*, $C'$, and $C''$ have no common crossing, then $C''$ separates the two crossings of *C* and $C'$.

Grünbaum [10] calls arrangements with this property ‘symmetric’. In the context of oriented matroids the property is part of the definition of arrangements of pseudocircles [2]. In [9] we call arrangements with this property “arrangements of great-pseudocircles” as they generalize the properties of arrangements of great-circles.

Arrangements of pseudolines which maximize the number of triangles have been studied intensively. Blanc [3] gives tight upper bounds for the maximum both in the Euclidean and in the projective case and constructs arrangements of pseudolines with $$\frac{2}{3}\left( {\begin{array}{c}n\\ 2\end{array}}\right) - O(n)$$ triangles for every *n*. In particular, for $n \equiv 0,4 \pmod 6$ projective arrangements of straight lines with $$\frac{2}{3}\left( {\begin{array}{c}n\\ 2\end{array}}\right) $$ triangles are known; see also [5]. This directly translates to the existence of (1) intersecting arrangements of pseudocircles with $$\frac{4}{3}\left( {\begin{array}{c}n\\ 2\end{array}}\right) - O(n)$$ triangles for every *n* and (2) intersecting arrangements of circles with $$\frac{4}{3}\left( {\begin{array}{c}n\\ 2\end{array}}\right) $$ triangles for $n \equiv 0,4 \pmod 6$. The ‘doubling method’ that has been used for constructions of arrangements of pseudolines with many triangles, see [3], can also be applied for pseudocircles. In fact, in the case of pseudocircles there is more flexibility for applying the method. Therefore, it is conceivable that $$\bigl \lfloor \frac{4}{3}\left( {\begin{array}{c}n\\ 2\end{array}}\right) \bigr \rfloor $$ triangles can be achieved for all *n*.

## Visualization

Most of the figures in this paper have been generated automatically. The programs are written in the mathematical software SageMath [13], they are available on demand. We encode an intersecting arrangement of pseudocircles by its dual graph. Each face in the arrangement is represented by a vertex and two vertices share an edge if and only if the two corresponding faces share a common pseudosegment. Note that, in the dual graph of every intersecting arrangement, the only 2-separators are the two neighbored vertices of a vertex corresponding to a digon. By replacing such digon-vertices by edges, we obtain a 3-connected graph which has the “same” embeddings as the original graph. Since 3-connected planar graphs have a unique embedding (up to isomorphism), the same is true for the original dual graph.

To visualize an intersecting arrangement of pseudocircles, we draw the primal (multi)graph using straight-line segments, in which vertices represent crossings of pseudocircles and edges connect two vertices if they are connected by a pseudocircle segment. Note that in the presence of digons we obtain double-edges.

In our drawings, pseudocircles are colored by distinct colors, and triangles (except the outer face) are filled gray. In straight-line drawings, edges corresponding to digons are drawn dashed in the two respective colors alternatingly, while in the curved drawings digons are represented by a point where the two respective pseudocircles touch.

### Iterated Tutte Embeddings

To generate nice aesthetic drawings automatically, we iteratively use weighted Tutte embeddings. We fix a non-digon cell as the outer cell and arrange the vertices of the outer cell as the corners of a regular polygon. Starting with edge-weights all equal to 1, we obtain an ordinary plane Tutte embedding.

For iteration *j*, we set the weights (force of attraction) of an edge $e=\{u,v\}$ proportional to $p(A(f_1)) + p(A(f_2)) + q(\Vert u-v\Vert /j)$ where $f_1,f_2$ are the faces incident to *e*, *A*(.) is the area function, $\Vert \cdot \Vert$ is the Euclidean norm, and *p*, *q* are suitable monotonically increasing functions from $\mathbb {R}^+$ to $\mathbb {R}^+$ (we use $p(x) = x^4$ and $q(x)=x^2/10$).

Intuitively, if the area of a face becomes too large, the weights of its incident edges are increased and will rather be shorter so that the area of the face will also get smaller in the next iteration. It turned out that in some cases the areas of the faces became well balanced but some edges were very short and others long. Therefore we added the dependence on the edge length which is strong at the beginning and decreases with the iterations. The particular choice of the functions was the result of interactive tuning. The iteration is terminated when the change of the weights becomes small enough.

### Visualization Using Curves

**Fig. 13:**
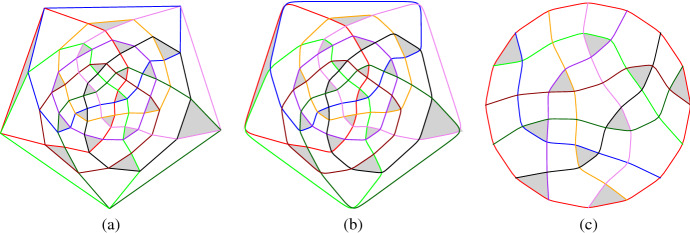
(**a**) Straight-line and (**b**) curved drawings of the arrangement of great-pseudocircles, which consists of two copies of (**c**) the non-Pappus arrangement of pseudolines

On the basis of the straight-line embedding obtained with the Tutte iteration we use splines to smoothen the curves. The details are as follows. First we take a 2-subdivision of the graph, where all subdivision-vertices adjacent to a given vertex *v* are placed at the same distance *d*(*v*) from *v*. We choose *d*(*v*) so that it is at most 1/3 of the length of an edge incident to *v*. We then use B-splines to visualize the curves. Even though one can draw Bézier curves directly with Sage, we mostly generated ipe files (xml-format, cf. [4]) so that we can further process the arrangements. Figure 13 (a) and (b) show the straight-line and curved drawing of an arrangement of pseudocircles, respectively.

### Visualization of Arrangements of Pseudolines

We also adapted the code to visualize arrangements of pseudolines nicely. One of the lines is considered as the “line at infinity” which is then drawn as a regular polygon. Figure 13 (c) gives an illustration.

### A More General Representation

**Fig. 14:**
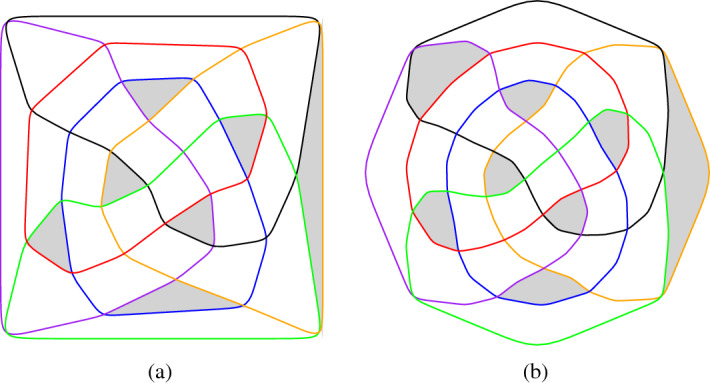
Two drawings of ${\mathscr {N}}_6^\Delta$: (**a**) curved primal graph. (**b**) curved primal-dual graph

As suggested in [9], intersecting arrangements with digons and non-intersecting arrangements of pseudocircles may be visualized by their primal-dual graph; see for example Fig. 14. Even though the primal-dual graph is a simple graph and has a unique embedding, we decided to stick to the above described visualizations because primal-dual graphs have about four times as many primitives as dual graphs and therefore are somewhat harder to read (for humans). In particular, *k*-cells in the arrangement are visualized as polygons of size 2*k* and therefore that representation is not that suitable for an article on cells. As an example consider the rightmost triangle bounded by the green, the orange, and the black pseudocircle in Fig. 14 (b) which actually looks like a quadrangle.

## Footnotes

1 There is a third type of move *take a hump* which is the inverse of “leave a pseudocircle”. However, this third type does not occur in the proof of Theorem 2.8 because each two pseudocircles already intersect.

2 While we only have a complete database of arrangements of up to seven pseudocircles (cf. Table 1 in [9]) we could make sure that there is no arrangement of eight pseudocircles with at least 39 triangles. Such an arrangement ${\mathscr {A}}$ would have a pseudocircle *C* such that the number of triangles of ${\mathscr {A}}' = {\mathscr {A}}- C$ would be at least 25. Our computations showed that no arrangement ${\mathscr {A}}'$ of seven pseudocircles with $p_3({\mathscr {A}}') \ge 25$ can be extended to an arrangement of eight pseudocircles with more than 38 triangles.
